# Effect of cooking treatments on the phytochemical composition and antidiabetic potential of *Vernonia amygdalina*

**DOI:** 10.1002/fsn3.732

**Published:** 2018-07-27

**Authors:** Florian Amel Tekou, Dieudonne Kuate, Philippe Tekem Nguekouo, Cerile Ypolyte Woumbo, Julius Enyong Oben

**Affiliations:** ^1^ Faculty of Science Department of Biochemistry Laboratory of Medicinal Plants Biochemistry Food Science and Nutrition University of Dschang Dschang Cameroon; ^2^ Faculty of Science Department of Biochemistry Laboratory of Nutrition and Nutritional Biochemistry University of Yaoundé 1 Yaoundé Cameroon

**Keywords:** antioxidant, culinary treatments, hypoglycemia, hypolipidemia, type 2 diabetes mellitus, *Vernonia amygdalina*

## Abstract

The present study aimed at evaluating the effects of domestic culinary treatment on phytochemical contents (phenolic content and dietary fiber), and the antidiabetic potential of *Vernonia amygdalina* in type 2 diabetic rats. The culinary forms implied boiling leaves of *V. amygdalina* directly and eliminating the leaves’ bitterness before boiling. Seventy wistar rats were artificially induced with type 2 diabetes using streptozotocin and high‐fat diet. They then received by oral intubation powders of different culinary forms of leaves extract or suspensions of *V. amygdalina* at a dose of 500 mg /kg for a period of 4 weeks. The crude fiber, total phenol contents and the DPPH scavenging ability of these culinary forms were also measured and the results showed that values of these parameters were higher in the unwashed form than the washed form. The washed and unwashed forms of *V. amygdalina* significantly reduced (*p* < 0.05) the blood glucose, the total cholesterol, triglyceride, transaminases, urea and creatine levels. Aqueous extract has the highest ability to reduce the blood glucose level (75.76%) followed by the unwashed form (61.17%) which was greater than that of the washed form. Also, these forms significantly increased serum HDL cholesterol and total protein level, with the highest activity obtained with the unwashed form. Washing the *V. amygdalina* leaves (that consists of multiple trituration of leaves with water) contributes to the reduction of antidiabetic and antioxidant properties.

## 1 INTRODUCTION

Type 2 diabetes is directly linked to obesity and accounts for 80% of diabetes cases (Webber, 2009). The number of adult suffering from diabetes has passed from 108 million in 1980–422 million in 2014, and recent estimates indicate more than 435 million of persons will be diabetic by 2030 if nothing is done (Webber, 2009). Many strategies have been put in place to manage this disease; these include the use of chemicals drugs and hypocaloric diets. Hypocaloric diets are slow in producing desired results while drugs, besides their side effects, are expensive (Kuate, Kengne, Biapa, Azantsa, & Muda, 2015; Woumbo, Kuate, & Womeni, 2017). This explains the renewed interest on research in functional foods for the diabetes management. In addition, epidemiological studies and clinical trials have shown that dietary intake of vegetables is correlated with a reduced risk of development of chronic diseases such as cancer, type 2 diabetes, and others (Woumbo et al., 2017).

*Vernonia amygdalina*, a member of the Asteraceae family, is a small shrub that grows in the tropical Africa. It is commonly called “bitter leaf” because of its bitter taste. The bitterness can, however, be abated by boiling or by soaking the leaves in several changes of water (Mwanauta, Mtei, & Ndakidemi, 2014). Previous studies have indicated that *V. amygdalina* leaves possess many properties such as hypoglycemic, hypolipidemic and antioxidant, due to their content in bioactive phytochemicals capable of disturbing the normal metabolism and helping in the management of diabetes (Adesanoye et al., 2016; Atangwho, Egbung, Ahmad, Yam, & Asmawi, 2013; Mwanauta et al., 2014; Ong, Hsu, Song, Huang, & Tan, 2011; Tonukari, Avwioroko, Ezedom, & Anigboro, 2015). Tonukari et al. (2015) showed that *V. amygdalina* contains many phytochemicals, including saponins, sesquiterpenes, lactones and flavonoids, steroid glucosides. *V. amygdalina* may also provide antioxidant benefit and it aqueous extract has been found to have antihelmintic, antitumor, hypoglycemic and hypolipidemic activities (Mwanauta et al., 2014 *)*. However, *V. amygdalina* leaves are never eaten raw, they always, like many others legumes and vegetables undergo culinary processes which can affect their bioactive phytochemicals content thus reducing their functional properties (Irondi, Akintunde, Agboola, Boligon, & Athayde, 2017; Mbondo, Owino, Ambuko, & Sila, 2018). In the cooking process of *V. Amygdalina*, leaves are commonly “washed” (a process that consists of multiple trituration with water) before eating to get rid of the bitter taste. Consistent with this, this work was thus designed to evaluate the effects of “washing” on phytochemical contents of *V. amygdalina* leaves, in relation to their antioxidant and antidiabetic potential.

## 2 MATERIALS AND METHODS

### 2.1 Plant material and reagent

Leaves of *V. amygdalina* were harvested from the farm of the University of Dschang in the West region of Cameroon, identified at the National Herbarium in Yaoundé and transported to the Laboratory of Medicinal Plants Biochemistry, Food Science and Nutrition of the Biochemistry Department of the University of Dschang where they were distributed into several portions to obtain the different culinary forms. Streptozotocin was purchased from A.G. Scientific, San Diego, CA, USA. All other chemicals with the exception of the kits were from Sigma‐Aldrich, St. Louis, MO, USA.

### 2.2 Aqueous extract and culinary forms

Aqueous extract of *V. amygdalina* leaves were obtained by soaking powders of corresponding leaves in water for 24 hr with gentle stirring, after which the mixtures were filtered using a Whatman N^o^4 filter paper. The resulting filtrates were dried at 45°C in an air oven to obtain the aqueous extract. To obtain the unwashed form powder, 100 g of clean leaves were cooked in 450 ml of water for 25 min at 95°C and dried at 45°C using an air oven and they were finally ground before administering to animals. The washed form powder was obtained using the same procedure described for the unwashed form powder with the only difference that before cooking, leaves were highly triturated with water until total elimination of their bitterness. The juice (which was not cooked and dried) was obtained from the filtrate after trituration of leaves in the presence of water. Cooking was done using stainless‐steel pot and ladle. Dried extracts and powders were sealed using Aluminum foil, stored in a desiccator and used daily to prepare the suspensions administered to animals. Powders were weighed as to theoretically give an equivalent extract mass utilized for aqueous extract, based on the extraction yield.

### 2.3 Phytochemicals and functional properties

#### 2.3.1 Crude fiber

Aqueous extract and powders of all *V. amygdalina* culinary forms were analyzed for crude fiber content using the *Ceramic Fiber Filter* as described by AOAC (1990, 1990). These extracts and powders were previously treated to remove lipids using hexane (24 hr soaking of 6 g of extracts and powders in 30 ml of hexane with gentle stirring). Briefly, 100 ml of 1.25% H_2_SO_4_ was added to 1 g of lipid free powder in a round bottom flask and the mixture boiled under reflux for 30 min. The hot solution was quickly filtered under suction. The insoluble matter was washed several times with hot distilled water until it was acid free. It was quantitatively transferred into the flask and 100 ml of hot 1.25% sodium hydroxide (NaOH) solution was added and the mixture boiled again under reflux for 30 min before it was quickly filtered under suction. The soluble residue was washed with boiling water until it was base free. It was then dried to a constant weight in the oven at 105°C, cooled in a desiccator and weighed. The weighed sample (C1) was incinerated in a muffle furnace at 300°C for about 2 hr, cooled in the desiccator and reweighed (C2). The loss in weight of sample on incineration was given by C1–C2 while the crude fiber content was expressed as follows:

$$\%\;\text{crude fibre}=\frac{C1-C2}{\text{Weight of original sample}}\times 100$$

#### 2.3.2 Phenolic content

Aqueous extract and powders of all *V. amygdalina* culinary forms were analyzed for total phenolic content using the Folin‐Ciocalteu method as described by Gao, Ohlander, Jeppsson, Björk, and Trajkovski (2000). Respectively 0.44 ml and 0.02 ml of distilled water and Folin reagent were added to 0.02 ml of extract/suspension of the culinary form of *V*. *amygdalina* (2 mg/ml) and allowed for 3 min, on to this was added 0.4 ml of 20% Na_2_CO_3_. The mixture was vortexed and incubated for 20 min at 40°C using a water bath, thereafter the absorbance was read against a blank at 760 nm using a BioMate 6 UV‐VIS spectrophotometer (BIOMATE). The total phenolic content was determined using the standard curve (*y* = 0.022 x; *r* ^2^ = 0.9945) obtained with Gallic acid. The contents were expressed as mg of Gallic Acid Equivalent/g of extract/powder.

#### 2.3.3 DPPH reducing ability of samples

The ability of the *V. amygdalina* leaves extract and suspensions of culinary forms to reduce the DPPH radical were tested as described by Mensor et al. (2001).

Briefly, 100 μl of extract/powders’ suspension was added to 900 μl of DPPH reagent (0.3 mM DPPH (2,2‐Diphényl‐1‐picrylhydrazyl) solubilised in methanol). After 30 min of incubation at room temperature, the absorbance was read at 517 nm against a blank; Butylhydroxytoluène (BHT) was used as standard.

### 2.4 Experimental animals and diets

Three‐week‐old Wistar rats were obtained from the Department Animal Centre and maintained in accordance with the guidelines of the OECD (2008). Thereafter, they were randomly distributed into seven groups of ten animals each (including two controls). The animals were individually housed under controlled temperature (25°C), lighting (12:12‐hr light–dark cycle) and had free access to water and diet. The test groups and the positive control were fed a high fat, high sucrose diet (17.6% fat and 7% sucrose‐enriched) while the negative control received a basal diet. The high‐fat‐high‐sucrose (HFHS) diet induced obesity was carried out for twelve (12) weeks. HFHS groups (*n* = 60) were fed a diet containing corn flour (52.9%), fish flour (20%), beef tallow (17.6%), bone (1%), vitamins (0.5%), salt (1%), sucrose (7%) while the negative control (BD) (n = 10) were fed a basal diet composed of: corn flour (77.8%), fish flour (20%), bone (0.1%), palm olein (1%), vitamins (0.1%), salt (1%). HFHS fed animals had free access to a 2% sucrose solution and the rats with a Body Mass Index (BMI) ≥ 0.68 g/cm^2^ were considered obese (Novelli et al., 2007).

These obese animals were administered with a single dose of 40 mg/kg of streptozotocin (Sigma Chemical Co). Three days after the injection of streptozotocin, fasting animals having a glycemic level higher than 200 g/L were considered as diabetics (Chang, 2000). The treatment lasted for 28 days and animals were grouped according to the treatments received as follows: unwashed form, washed form, juice, aqueous extract, metformin, and water for the two controls groups. Treatments were administered once a day by oral intubation between 8 a.m. and 11 a.m. local time with water as vehicle for both extracts and suspensions at the dose of 500 mg/kg. All experiments were carried out according to the regulations and ethical approval of the Experimental Animal Welfare and Ethics Committee of the Institution.

### 2.5 Biological parameters: Blood Glucose level and Serum lipids, transaminases, total protein, urea, and creatinine

The blood glucose (expressed in mg/dl) was measured (5–10 μl from tail tip) after an overnight fasting (8 hr), using a portable glucometer (Accu‐Chek) every weeks during the treatment.

The lipid profile was determined using colorimetric methods (MONLAB kits) using the standard protocols described by Trinder (1969). For the total cholesterol, Huang, Kao, and Tsai (1997). For HDL cholesterol and LDL cholesterol was estimated using the formula established by Friedewald, Levy, and Frederickson (1972).

Calorimetric methods (IMESCO kits) were also used to determine the serum level of alanine aminotransferase, aspartate aminotransferase. The standard protocol described by Lowry, Rosbrough, Farr, and Randall (1951) was used to determine total protein as well as Bartels, Böhmer, and Heierli (1972) for Creatinine and Marshall (1913) for urea.

### 2.6 Statistical analysis

Statistical analysis was performed using SPSS program version 21. Results were expressed as mean ± standard deviation (*SD*). One‐way analysis of variance (ANOVA) with Bonferroni test was used for statistical analysis of the mean difference among groups. Differences were considered significant at *p* < 0.05 (at 95% confidence interval).

## 3 RESULTS AND DISCUSSION

### 3.1 Phytochemical contents and antioxidant potentials

#### 3.1.1 Crude fiber

**Figure 1:**
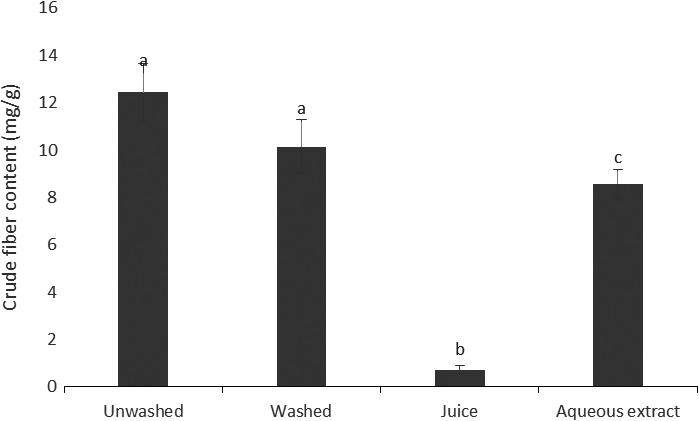
Crude fiber content of aqueous extract and powders of *Vernonia amygdalina* culinary forms. Values with different letters are significantly different at *p* < 0.05

Figure 1 shows the crude fiber content of aqueous extract and powders of *V. amygdalina* culinary forms obtained by the *Ceramic Fiber Filter* (A.O.A.C). The unwashed form exhibited the highest fiber content (12.43 mg/g). This could be explained by the fact this form did not undergo trituration. Consequently, only a few losses in fiber occurred. We also noticed that there was a very limited amount of crude fiber in juice, and this could be due to the different food treatments used. So these results show that washing *V. amygdalina* leaves before cooking reduces its crude fiber.

#### 3.1.2 Total phenolic contents

**Figure 2:**
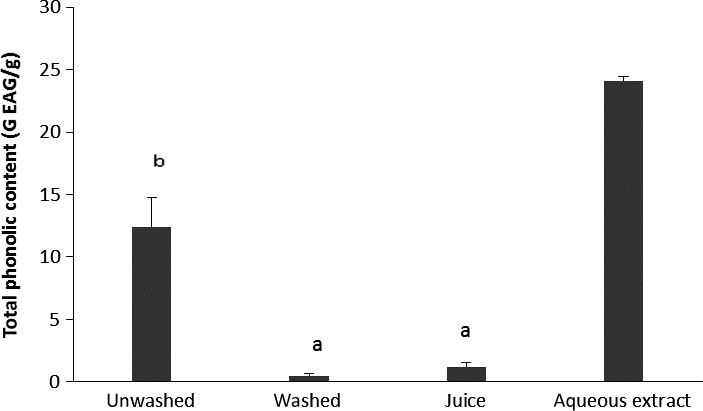
Phenolic contents of aqueous extract and powders of *Vernonia amygdalina* culinary forms. Values with different letters are significantly different at *p* < 0.05

Phenolic compounds constitute an important class of compounds in the action of functional foods; their antioxidant properties are responsible of the reduction of complications associated with diabetes either directly or indirectly. Therefore, it was important for us to find out the impact of cooking process on their contents in foods. Figure 2 shows the variation of phenolic contents expressed in mg of Gallic Acid Equivalent/g of aqueous extract and powders of all *V. amygdalina* culinary forms. It appears that the phenolic content ranges from 24.08 mg EAG/g for the aqueous extract to 0.445 mg EAG/g for the washed form. The observed differences could be explained by the methods used to obtain our culinary forms. So, aqueous extraction had the highest level of phenolic compound, while washing *V. amygdalina* leaves before cooking reduces its total phenolic contents.

#### 3.1.3 DPPH scavenging ability

**Figure 3:**
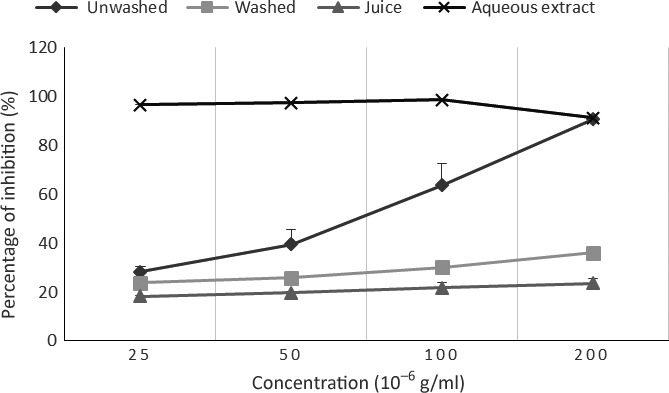
Change in the antioxidant activity of aqueous extract and powders of *Vernonia amygdalina* culinary forms at different concentrations

The ability of our samples to reduce free radicals was evaluated as DPPH scavenging activity because trapping the reactive oxygenated species and free radicals constitute one of the mechanisms of action (the most important) of antioxidants. So DPPH form a stable molecule when accepting a proton or an electron, and therefore is used in the determination of trapping effect of radicals in natural products (Marfak & Sanchez, 2002). Figure 3 presents the profile of the antioxidant activity of aqueous extract and powders of all *V. amygdalina* culinary forms at different concentrations. The highest activity was obtained for the aqueous extract while the juice presented the lowest antioxidant activity. We also observed the same trends regarding the reducing ability and the phenolic content; so this result can be attributed to the presence of phenolic compounds in our different samples, as shown by Pietta (1998) and Katalinic, Milos, and Jukic (2006)

### 3.2 Blood glucose level during the treatment

**Figure 4:**
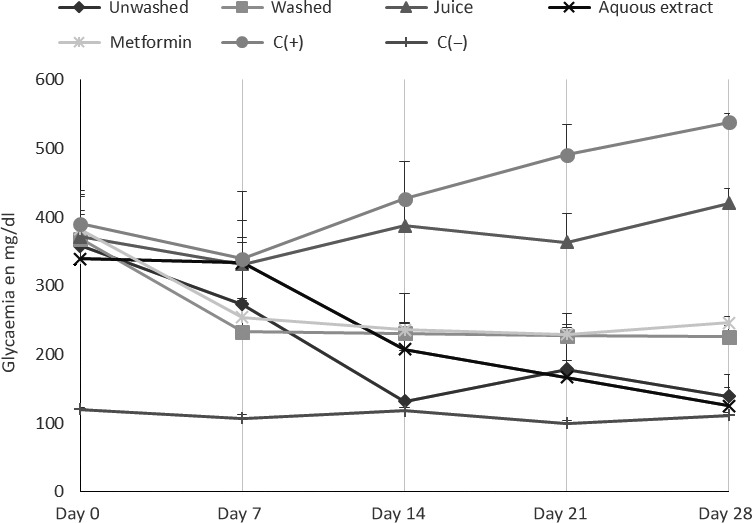
Changes in blood glucose concentrations during the treatment

Figure 4 shows the fasting blood glucose concentrations taken at seven‐day intervals during 28 days of treatment. It appears that at the 28th day, the glycaemia of positive's control was significantly higher than those of negative's control; these could be explained by the effectiveness of disease on those diabetic rats. It also resulted that aqueous extract exhibited the highest ability to reduce the blood glucose level (75.76%) followed by the unwashed form (61.17%); this could be explained by the presence in high quantities of dietary fiber and phenolic compounds. In fact, phenolic compounds have the ability to inhibit alpha amylase (McCue, Kwon, & Shetty, 2005) thus inducing the reduction of glycaemia. On the other hand, dietary fiber inhibits glucose absorption by limiting its intestinal absorption (Klosterbuer & Slavin, 2010).

### 3.3 Effect of the treatments on the lipid profile

**Figure 5:**
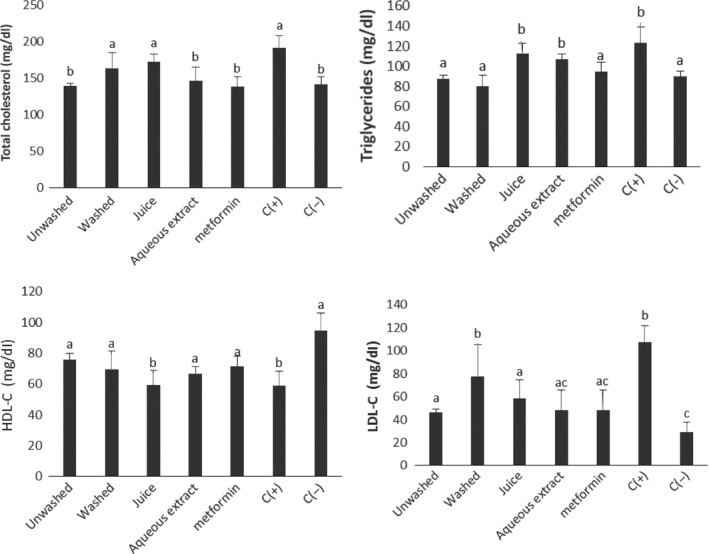
Serum concentration of triglyceride, total cholesterol, HDL and LDL cholesterol in animals after 28 days of treatment. Values with different letters are significantly different at *p* < 0.05

Figure 5 depicts the serum concentration of triglyceride, total cholesterol, HDL and LDL cholesterol of animals after 28 days of treatment. The mechanism by which aqueous extract and powders of *V. amygdalina* culinary forms exert their activity on lipid profile is not clearly detailed, but on this figure, we can observe that the unwashed form had the highest ability to reduce the serum level of LDL cholesterol; this result could be attributed to the presence of compounds with high hypolipidemic property especially dietary fiber and phenolic compounds as shown by Nsor‐Atindana, Zhong, and Mothibe (2012). Also, the highest ability to reduce the serum level of triglycerides was obtained from animals that had received powders of unwashed form of *V. amygdalina* leaves. Diabetes usually results in an increase in the activity of lipases which is responsible of lipid metabolism disorders. Thus, this powders’ ability of the unwashed form of *V. amygdalina* leaves to reduce the level of triglycerides could be attributed to bioactive compounds. These results corroborate with those of Espindola et al. (2016) who had shown that phenolic compounds have the ability to reduce the serum level of triglycerides.

With regard to the HDL cholesterol, we obtained an increase in the serum level of HDL cholesterol in animals that received aqueous extract and powders of *V. amygdalina* culinary forms with the exception of those taking the juice of *V. amygdalina*. The highest increase in HDL cholesterol was obtained with the unwashed form of *V. amygdalina* leaves. These results corroborate with those of Asante et al. (2016) who previously showed that phenolic compounds have the ability to increase the serum level of HDL cholesterol.

### 3.4 Effect of treatments on serum transaminases and total protein

The hyperglycemia caused by diabetes is responsible for many complications of the disease.

**Figure 6:**
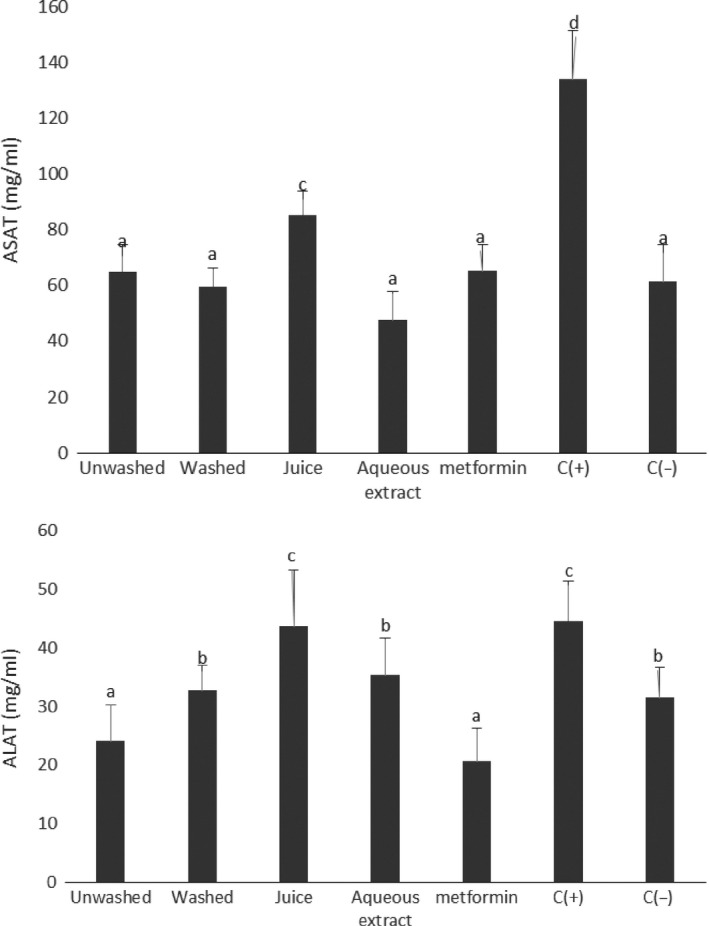
serum level of alanine aminotransferase (ALAT), aspartate aminotransferase (ASAT) and total protein. Values with different letters are significantly different at *p* < 0.05

Liver is the central organ involved in the metabolic processes of organism, and is affected by diabetes complications. In this work, we tried to find out if the aqueous extract and powders of *V. amygdalina* culinary forms protect against or have the capacity to reduce the effects of diabetes complications in the liver as an increase in the transaminase activities has been shown to cause lesions in this organ (Hultcrantz, Glaumann, & Lindberg, 1986). Figure 6 shows the serum level of alanine aminotransferase (ALAT), aspartate aminotransferase (ASAT) and total protein of animals being treated with aqueous extract and powders of *V. amygdalina* culinary forms. With the exception of the juice of *V. amygdalina* leaves, all the samples showed an ability to reduce the serum level of the transaminases and to increase the total protein level. Similar results were obtained by Adesanoye et al. (Adesanoye et al., 2016) who demonstrated that methanolic extract of *V. amygdalina* has the ability to reduce the activity of serum transaminases.

### 3.5 Effect of treatments on serum urea and creatinine

**Figure 7:**
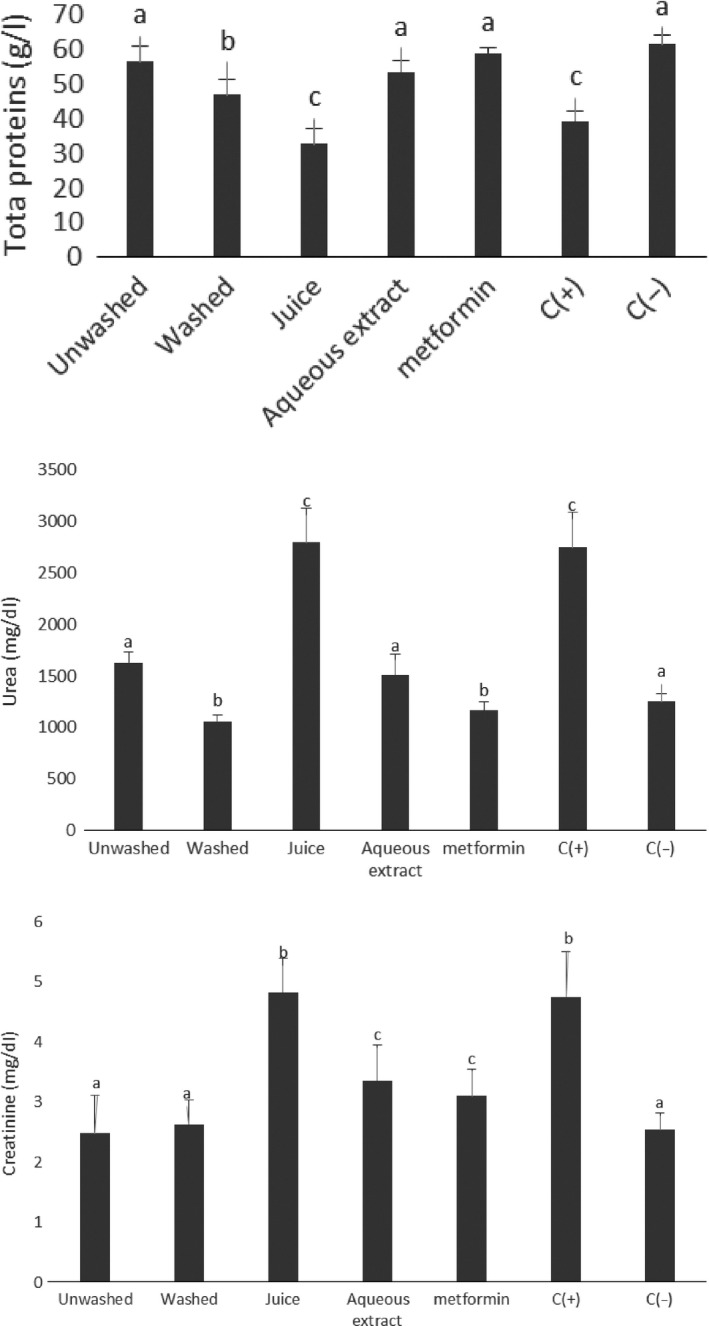
serum level of urea and creatinine. Values with different letters are significantly different at *p* < 0.05

Diabetes complications also cause nephropathy. In fact, kidney is an organ which maintains the hydroelectric equilibrium of the organism. It is responsible of the eliminations of metabolic wastes, such as urea (product of protein catabolism in the body) and creatinine (product of creatine catabolism in the muscle) (Boubchir, 2002). So a high level of these compounds in the serum could reflect in a problem at the level of kidneys. In this study, the level of urea and creatinine was significantly higher for positive than negative control (Figure 7). The highest ability to reduce the serum level of urea and creatinine was obtained from animals receiving powders of the washed form of *V. amygdalina* leaves and powders of the unwashed form of *V. amygdalina* leaves, respectively. The powder of the washed form of *V. amygdalina* leaves had lowest level of phenolic compounds, so its activity could be attributed to dietary fibers. Then the ability of unwashed form of *V. amygdalina* leaves to regulate the level of creatinine could be explained by its high level of dietary fiber and phenolic compounds.

## 4 CONCLUSION

In conclusion, the different culinary treatments applied to *V. amygdalina* leaves before eating have a great impact on their composition and their functional properties. So cooking *V. amygdalina* leaves reduces its level of bioactive compounds. We have also observed a low ability of “washed leaves” to correct disorders due to diabetes. Thus, washing the *V. amygdalina* leaves (which consists of multiple trituration of leaves with water) contribute to the reduction of antidiabetic and antioxidant properties.

## CONFLICT OF INTEREST

the authors declare no conflict of interest.

## ETHICAL REVIEW

Animals were maintained in accordance with the guidelines of the OECD (2008). All experiments were carried out according to the regulations and ethical approval of the Experimental Animal Welfare and Ethics Committee of the Institution.
